# Classification and Locoregional Treatment of Rectal Neuroendocrine Tumors

**DOI:** 10.7759/cureus.40128

**Published:** 2023-06-08

**Authors:** Gurdeep Singh, Arooj Mian, Mehreen Ali, Swotantra Gautam, Aimen Farooq

**Affiliations:** 1 Internal Medicine, AdventHealth Orlando, Orlando, USA

**Keywords:** malignancy, endoscopic mucosal resection, gastroenterology, endoscopy, rectal neuroendocrine tumor

## Abstract

A 43-year-old male presented to his primary care physician’s office with a complaint of painless rectal bleeding with a concomitant weight loss of 10-15 pounds and intermittent abdominal pain. Endoscopic evaluation was remarkable for a 5 mm rectal polyp roughly 10 cm from the anal verge. Resection was performed and the pathology was consistent with a low-grade neuroendocrine/carcinoid tumor. Immunostaining for synaptophysin, chromogranin, CD56, and CAM5.2 were positive while staining for CK20 was negative. Given the absence of metastasis on radiographic and endoscopic evaluation, the patient was managed conservatively thereafter with observation. Despite having an indolent clinical course, resection is recommended for all rectal neuroendocrine tumors. Locoregional endoscopic resection versus radical resection can be used for adequate tissue removal depending on the characteristics of the tumor and the degree of invasion.

## Introduction

Gastrointestinal neuroendocrine neoplasms (NENs) are a rare clinical entity that arises from the dysregulated proliferation of neuroendocrine cells in the digestive tract [1]. The World Health Organization has subdivided NENs into neuroendocrine tumors (NETs) and neuroendocrine carcinomas (NECs), with the dichotomy primarily stemming from the degree of differentiation and proliferative activity [1]. Poorly differentiated neuroendocrine neoplasms are called NECs and well-differentiated neoplasms are called NETs [1]. NENs can be functionally active or inactive depending on their ability to secrete bioactive peptides [2].

The gastroenteropancreatic system is by far the most common site of neuroendocrine tumorigenesis with 70% of NENs arising from there [1]. Rectal NETs represent 1-2% of all rectal tumors and generally follow a benign course [1,3]. The annual incidence of rectal NETs in the United States is 0.93/100,000 and their prevalence is 5.1/100,000 [3]. Rectal NETs are generally diagnosed through tissue sampling and subsequent histopathological analysis. The management of rectal NETs often involves a multidisciplinary approach, with treatment modalities varying depending on the clinical characteristics of the tumor, the degree of local invasion, and the presence of local or distal metastasis [2]. Five-year survival is 87% in localized disease and roughly 25% in metastatic disease [3].

## Case presentation

A 43-year-old male with a past medical history of hypertension, obesity, and obstructive sleep apnea presented to his primary care physician’s office with complaints of painless rectal bleeding with a concomitant weight loss of 10-15 pounds and intermittent abdominal pain. He was referred to Gastroenterology and underwent a diagnostic colonoscopy. Endoscopic evaluation was remarkable for internal hemorrhoids and a 5 mm rectal polyp roughly 10 cm from the anal verge. The polyp was Paris classification IIa, was lifted with a Voluven, indigo carmine, and epinephrine 1:100,000 injection, and was resected using a hot snare.

**Figure 1:**
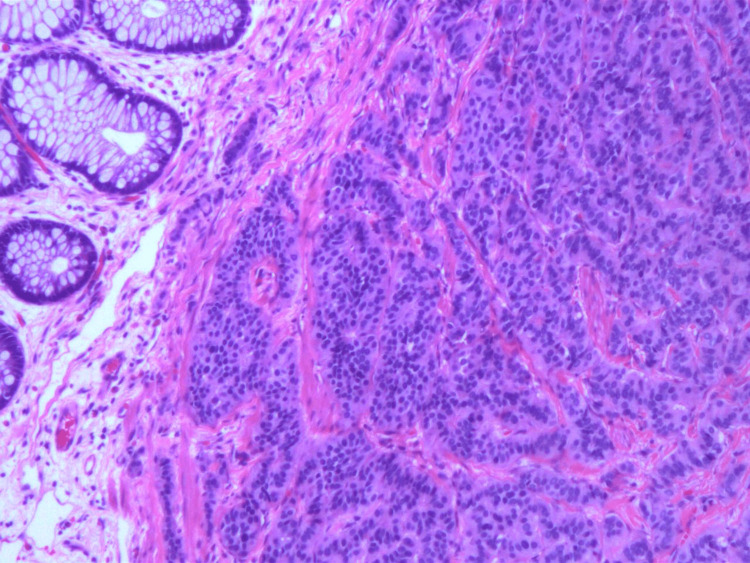
Histopathology showing mucosal glands overlying the submucosal carcinoid tumor

**Figure 2:**
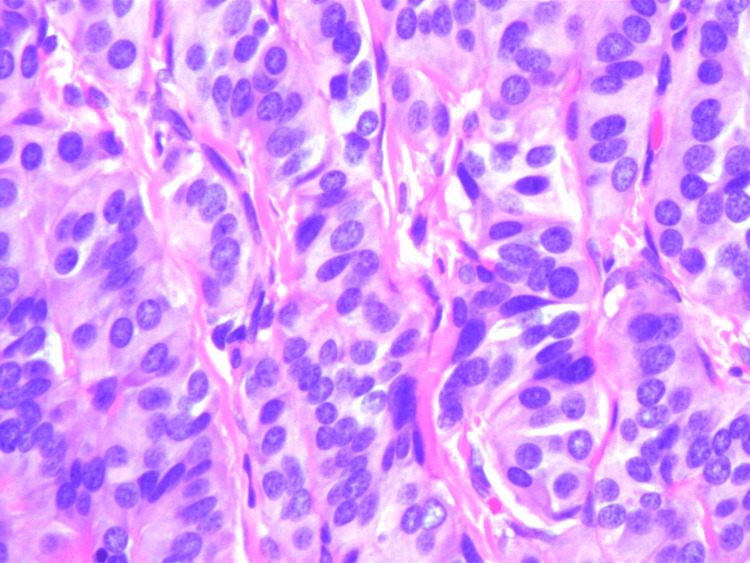
High-power view showing nests and cords of cells with monomorphic nuclei and stippled chromatin

**Figure 3:**
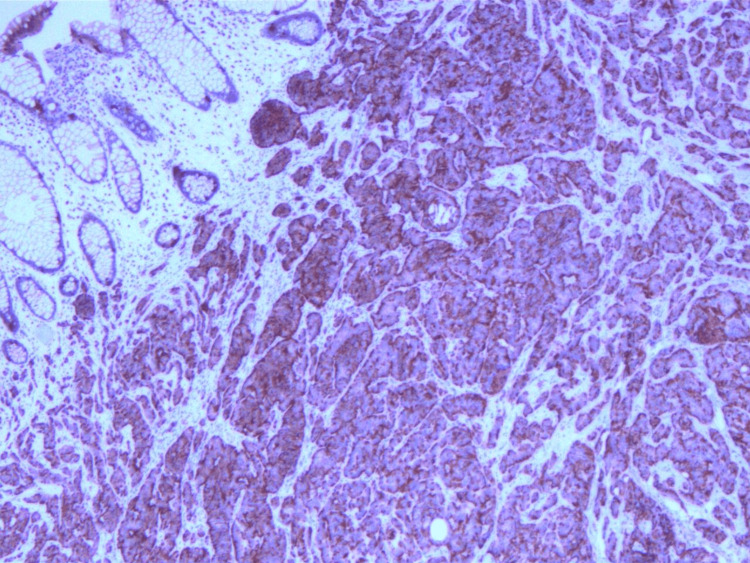
Immunohistochemical staining of slide demonstrating positivity to chromogranin

**Figure 4:**
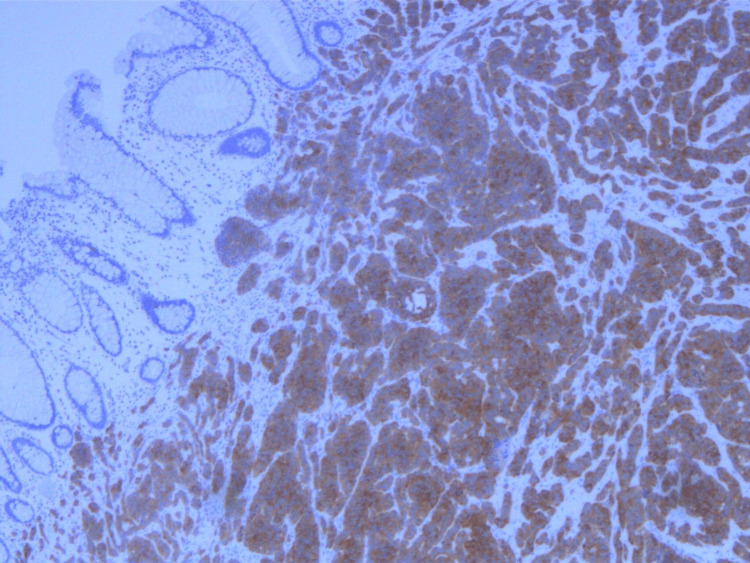
Positive immunohistochemical staining for synaptophysin

The rectal tissue specimen was sent for histopathologic analysis and was determined to have staining characteristics consistent with a low-grade neuroendocrine/carcinoid tumor. Sections demonstrated unremarkable rectal mucosa overlying a submucosa, with nests and cords of cells showing monomorphic nuclei and stippled chromatin (Figures 1, 2). The peripheral margins appeared free of tumor burden. Immunostaining for chromogranin (Figure 3), synaptophysin (Figure 4), CD56, and CAM5.2 was positive while staining for CK20 was negative. The proliferative marker MIB-1 demonstrated less than 3% immunopositivity, supporting the low-grade nature of the tumor.

The patient underwent a subsequent lower endoscopic ultrasound (EUS) that revealed localized wall thickening at the site of endoscopic mucosal resection that appeared to be primarily due to focal thickening of the muscularis propria. However, no residual lesions or lymph nodes were noted in the perirectal region.

The patient was referred to Hematology/Oncology for further evaluation and management. A CT scan of the abdomen/pelvis was ordered and revealed no metastatic disease. Given the absence of metastasis on radiographic and endoscopic evaluation, the patient was managed conservatively with observation. Subsequent CT scans of the abdomen/pelvis continued to remain negative for distal organ or lymphovascular metastases. He underwent a repeat colonoscopy in 2020 that revealed only a hyperplastic polyp in the rectum. Repeat lower EUS was also unremarkable and did not reveal disease recurrence. Serial serum Chromogranin A levels remained within normal limits. The patient is presently in good health and continues to follow up with Hematology/Oncology and Gastroenterology for periodic evaluation.

## Discussion

Rectal neoplasia can be categorized as epithelial or neuroendocrine in etiology [2]. Epithelial rectal neoplasms include adenocarcinomas, squamous cell carcinomas, and mixed-adenosquamous carcinomas [2]. In 2010, the World Health Organization released a classification system for gastrointestinal neuroendocrine neoplasms. NENs were broken down into neuroendocrine tumors, neuroendocrine carcinomas, and mixed neuroendocrine non-neuroendocrine tumors (MiNENs) [2]. NETs are considered well-differentiated NENs while NECs are considered poorly differentiated. However, there is heterogeneity present within NETs depending on the proliferative rate of the tumor [2]. Grade 1 (G1) NETs have a mitotic rate of < 2/10 high power field (HPF), or a Ki-67 proliferation index < 2% [2]. G2 tumors have a mitotic rate of 2-20/10 HPF or a Ki-67 proliferation index between 2% and 20% [2]. Well-differentiated NENs classified as G3 demonstrate a mitotic rate > 20/10 HPF or a Ki-67 proliferation index greater than 20% and are considered NECs [2]. Within the rectum, well-differentiated tumors represent the vast majority of NENs [2]. Gastrointestinal neuroendocrine tumors generally arise from chromaffin cells or Kulchitsky cells that line the epithelium of the digestive tract and originate in the crypts of Lieberkühn [4]. The incidence of rectal neuroendocrine tumors has been increasing over the last several decades, likely due to increased access to screening colonoscopies [4].

Clinical manifestations for patients with rectal NETs can vary, with roughly 50% of patients being asymptomatic at the time of discovery [5]. The most common complaint in symptomatic patients was anorectal discomfort followed by rectal bleeding [4]. Other symptoms include constipation, change in bowel habits, weight loss, abdominal pain, and diarrhea [4]. The vast majority of rectal NETs are functionally inactive and produce more glycine and glucagon relative to their enteric NET counterparts that produce greater quantities of serotonin [4].

Rectal NETs generally follow an indolent course, often leading to the misconception that these are benign tumors. However, they remain malignant entities and retain metastatic potential. Historically, tumor size was used to stratify patients with rectal NETs with tumors less than 10 mm in size having low metastatic potential, tumors between 10 and 20 mm in size having intermediate metastatic potential, and tumors greater than 20 mm having a relatively higher overall risk of metastasis [4]. This echoes the findings by McDermott et al.’s meta-analysis of 4,575 patients with rectal NETs, as they found tumor sizes greater than 10 mm portended a higher risk of metastatic disease [6]. Muscular and lymphovascular invasion were also independent risk factors associated with metastasis [6]. Naunheim et al. examined tumors less than 20 mm in size and found that the risk of metastasis was 2% if confined to the submucosa but rose to 46% if there was muscular involvement [7].

The diagnosis and treatment of rectal neuroendocrine tumors hinge on adequate tissue sampling and subsequent immunohistopathologic analysis. Localized treatment options for rectal NETs include endoscopic modalities like polypectomy, endoscopic mucosal resection (EMR), advanced endoscopic resection, transanal endoscopic microsurgery, or cap-assisted EMR as well as surgical options, including transanal surgical resection, low anterior resection, or abdominoperineal resection [8]. Observation alone has led to inferior outcomes, particularly with regard to patient mortality [8]. In Zhao et al.’s multivariable Cox regression analysis, they found no significant differences in five-year survival between local resection and radical resection, but observation alone did lead to a worse overall survival at five years (HR 2.750, p < 0.001) [8].

Optimal therapeutic options depend on the characteristics of the tumor, the persistence of disease at the margins, and overall disease progression at the time of discovery. Kwaan et al. reviewed 85 patients with rectal NETs and discovered that 38 out of 48 tumors less than 1 cm in size were found to have positive or indeterminate margins in the histological examination after endoscopic resection [9]. Of these 38 tumors, six were found to have recurrent disease on subsequent endoscopy and one was found to have metastatic disease. Based on their findings, they recommended transanal resection if tumors were found to have positive margins at the time of resection irrespective of tumor size, high-risk features, or local invasion into the muscularis propria. Transanal excision was also recommended for tumors between 1.0 and 1.9 cm in size without high-risk features [9]. Their recommendations contrast with that of Kim et al. who discovered that 107 tumors less than 10 mm in size that underwent endoscopic resection did not have any evidence of disease recurrence or metastasis at the time of follow-up, irrespective of margin status, as roughly 50% of the tumors sampled had indeterminate or positive margins at the time of histological analysis [10]. Radical resection is recommended for NETs between 10 and 19 mm in size that demonstrate evidence of muscular invasion, have high-grade features, or that are larger than 20 mm in size, a recommendation that falls in line with the guidelines from the National Comprehensive Cancer Network and European Neuroendocrine Tumor Society [11,12]. 

The role of excisional therapy has been debated for tumors between 1.0 to 1.9 cm without high-risk features or muscular involvement, as rates of metastasis have been considered to be roughly 10-15% [12]. The utility of novel endoscopic techniques over the last several decades such as endoscopic submucosal dissection (ESD) was explored in Chen et al’s retrospective analysis of tumors < 20 mm in size [13]. They found that 90.4% of patients had effectively resected tumors with a 3.35% risk of complications, the latter of which should downtrend in the coming years as endoscopists increase their familiarity with advanced endoscopic resection [13].

Overall, endoscopic polypectomy and standard EMR are considered inferior to modified EMR (m-EMR) or ESD at achieving en bloc resection based on a meta-analysis by Le et al. [14]. Their data reflected a higher rate of complete resection using ESD or m-EMR compared to standard EMR (OR = 0.42, 95% CI 0.25 - 0.72; OR = 0.10, 95% CI 0.03 - 0.33, respectively) [14]. No significant differences were noted between m-EMR and ESD with respect to resection rates or complications, but ESD was found to take a longer time to complete [14]. Within m-EMR, the rates of en bloc resection of EMR using a ligating device (EMR-L) and cap-assisted EMR (EMR-C) were compared in a retrospective analysis using 158 patients in a Korean tertiary care hospital [15]. They found that the en bloc resection rate was higher for EMR-L compared to EMR-C (100% vs 92.9%, p = 0.003) with no significant differences in procedural complications (p = 0.870) [15].

## Conclusions

Rectal NENs are divided into neuroendocrine tumors and neuroendocrine carcinomas. NETs differ from NECs based on their degree of differentiation and NETs are further subdivided according to their proliferative activity using mitotic rate and KI-67 index. Despite having an indolent clinical course, resection is recommended for all rectal neuroendocrine tumors. For tumors less than 10 mm in size without high-risk features or invasion into the muscularis propria, endoscopic resection is recommended. For tumors greater than 20 mm in size, surgical resection is the mainstay of treatment. While the optimal therapy for tumors between 10 and 20 mm in size is controversial, the evidence suggests radical resection for tumors with high-risk characteristics or local invasion and advanced endoscopic techniques like m-EMR or ESD for en-bloc resection in tumors without these features.
